# Kir4.1 and Aqp4 Contribution to Schisis Cystic Water Accumulation and Clearance in the Rs1 Exon-1 Del XLRS Rat Model

**DOI:** 10.3390/genes15121583

**Published:** 2024-12-09

**Authors:** Zeljka Smit-McBride, Ning Sun, Serafina Thomas, In Hwan Cho, Robin G. Stricklin, Paul A. Sieving

**Affiliations:** 1 Department of Ophthalmology, Eye Center, UC Davis School of Medicine, University of California Davis, Sacramento, CA 95817, USA; 2 Vitreoretinal Research Lab, UC Davis School of Medicine, University of California Davis, Davis, CA 95616, USA; 3 Department of Cell Biology & Human Anatomy, University of California Davis, Davis, CA 95616, USA; 4 Department of Ophthalmology, College of Medicine, Soonchunhyang University, Cheonan 31151, Chungcheongnam-do, Republic of Korea

**Keywords:** retinoschisin, RS1, aquaporin4, Aqp4, Kir4.1, X-linked retinoschisis, XLRS, rat retina disease model, deep capillary plexus, Muller glia cells, MGC, retina development

## Abstract

Background/Objective: The Rs1 exon-1-del rat (Rs1KO) XLRS model shows normal retinal development until postnatal day 12 (P12) when small cystic spaces start to form in the inner nuclear layer. These spaces enlarge rapidly, peak at P15, and then collapse by P19. Methods: We explored the possible involvement of Kir4.1 and Aqp4, the principal retina channels for water movement and homeostasis, along with Muller glia cells (MGCs), using semi-quantitative fluorescent immunohistochemistry at P7, P9, P12, and P30, in Rs1KO and WT littermates. Results: Kir4.1 expression was reduced in Rs1KO retinas at all the early time points—P7, P9, and P12—as the schisis cavities began to form; downregulation would reduce water egress from the retina. Aqp4 was upregulated at P30 in Rs1KO retinas during schisis cavity closure but not as cavities formed at P12. When examined by GFAP expression, MGCs were not activated at the preschisis P12 age but showed considerable GFAP expression at P30 following retinal cystic structural damage at P15, indicating that MGCs were activated during the period of retina water removal and cavity closure. Conclusions: The study results implicate the downregulation of Kir4.1 in schisis formation and a role for both Kir4.1 and Aqp4 upregulation in subsequent schisis closure.

## 1. Introduction

X-linked retinoschisis (XLRS) is a rare genetic disorder characterized by splitting of the layers within the macula of the retina, leading to visual impairment in affected males. The clinical phenotype of XLRS is broad and may be limited to the macula or also show peripheral retinal abnormalities [1]. XLRS prevalence is estimated at 1/5000 to 1/20,000 with onset usually in early childhood [2]. Diagnosis is based on observing macular cystic changes by clinical examination and confirmed with optical coherence tomography (OCT) imaging and electroretinogram (ERG) findings of a reduced scotopic ratio of b-wave to a-wave amplitudes. Genetic testing for RS1 mutations is available [3]. XLRS also affects the macular microvasculature, leading to decreased flow density in the deep capillary plexus, with slow loss of photoreceptors and microvascular alterations correlating with functional outcomes [4,5]. 

The RS1 gene encodes the protein retinoschisin, which is essential for cell–cell adhesion and the integrity of the retinal intracellular matrix [6]. The mechanisms of pathology are not fully understood, but in rough terms, RS1 is required for proper adhesion between retinal cells and to coordinate other protein moieties at the cell surface [7,8,9,10]. RS1 is also required to maintain synaptic function between photoreceptors and bipolar cells [11,12]. In the absence of RS1, homeostasis of intraretinal fluid appears to be altered and results in fluid accumulation as intraretinal schisis cystic spaces. 

We created an Rs1 exon-1-del rat (Rs1KO) XLRS model which shows normal retinal development by light microscopy up to postnatal day 12 (P12) when small cystic spaces begin to form in the middle of the inner nuclear layer (INL) [13]. These cysts then enlarge rapidly, and by P15, they double in INL thickness. Then, the cavities rapidly collapse by P19–P21. Fluid accumulation within the middle layer of the retina during this period implicates aberrant water homeostasis. 

**Figure 1:**
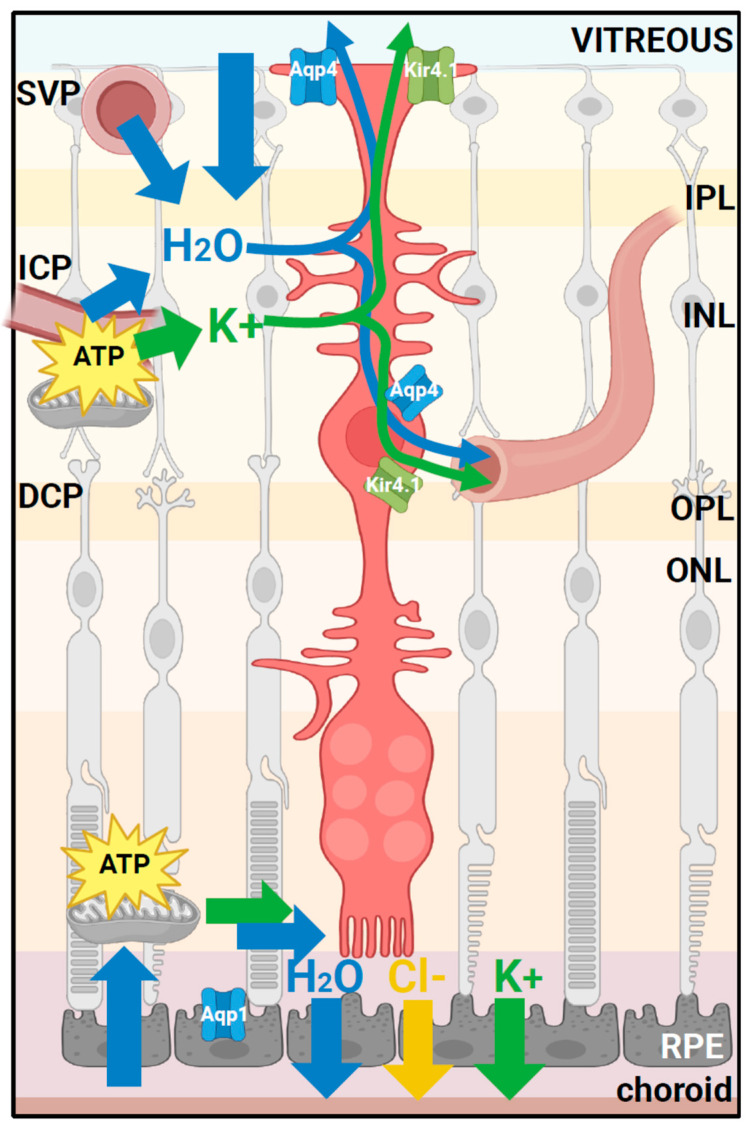
A schematic representation of a retinal Muller glial cell (MGC) illustrating its roles in ions and water drainage from the inner retina toward the retinal vessels. Potassium transport is associated with water drainage through inwardly rectifying potassium channels (Kir4.1) and aquaporin 4 (Aqp4) channels, both located close to the interface of the retinal MGC with retinal vessels and in retinal MGC endfeet at the level of the internal limiting membrane. Image created using BioRender: Smit-McBride, Z. (2024) https://BioRender.com/p88p075 (accessed on 25 November 2024). IPL, inner plexiform layer; INL, inner nuclear layer; OPL, outer plexiform layer; ONL, outer nuclear layer; RPE, retinal pigment epithelium; SVP, superficial vascular plexus; ICP, intermediate capillary plexus; DCP, deep capillary plexus; ATP, adenosine triphosphate.

Homeostasis of intraretinal fluid requires maintaining ionic balance mechanisms by Muller glial cells (MGCs) [14], which maintain the structural and functional integrity of the retina [15,16]. MGCs are highly polarized, with cell bodies in the INL and radial processes that extend across the entire retinal thickness (Figure 1). MGCs maintain a concentration of aquaporin 4 (Aqp4) water channels and inwardly rectifying potassium channels, particularly Kir4.1, at distinct MGC endfeet membrane domains of the inner limiting membrane (ILM) adjoining the vitreous. They also make contact with the three capillary plexuses within the retina: the superficial vascular plexus (SVP), the intermediate capillary plexus (ICP), and the deep capillary plexus (DCP) at the INL/OPL margin [17]. MGCs maintain the proper concentration of retinal extracellular K^+^ potassium ions [18]. This includes the uptake of K^+^ ions during neuronal activity and redistribution into vitreal, vascular, and subretinal spaces [15,19]. K^+^ uptake/redistribution is performed through a high density of inwardly rectifying potassium channels 4.1 (Kir4.1) on MGCs. The movement of K^+^ ions is coupled to water transport, which is further facilitated by the spatial distribution of Aqp4 water pores [20,21]. Kir4.1 channels also maintain the negative membrane potential of MGCs and other glial cells [15]. Hence, the functional expression of Kir4.1 channels in glial membranes plays a significant role in voltage-dependent transport processes, including glutamate uptake and signaling [22,23]. Glutamine synthetase (GS) is a key enzyme in glutamate signaling and is expressed exclusively by MGCs to remove active, neuron-released glutamate signaling transmitters by converting them into glutamine [24]. 

MGCs’ involvement in XLRS pathology has been suggested previously [15,25,26], but their mechanisms remain an open question. The timing of these observations is critical, as it is probable that MGCs are affected once XLRS disease pathology has occurred. Our question was whether the dysfunction of the homeostatic regulation of water and ion channels contributes to the initial formation of schisis cavities during retinal development and whether MGC activation coupled with swelling of MGC soma contributes to schisis formation at P12.

We closely monitored the retinal expression of Kir4.1 and Aqp4 during times P7, P9, and P12 prior to and just as the first schisis cavities form in this XLRS rat model. We also looked at the expression of glial fibrillary acidic protein (GFAP), a marker for MGC activation. We paid particular attention to whether MGC structural alterations or the expression and distribution of Aqp4 and Kir4.1 were altered by the time of initial cavity formation, as this would implicate these mechanisms in contributing to XLRS retinal pathology. 

We also looked at P30, which is after the maximal schisis cavity formation at P15, to observe structural and functional consequences resulting from the massive INL cysts which are mostly resolved by P19. We were interested in whether retinal stress-activated response mechanisms, including the upregulation and redistribution of Aqp4 and Kir4.1, result from the formation and rapid closure of schisis cavities. 

## 2. Materials and Methods

### 2.1. Animals

The animal experiments were conducted according to UC Davis IACUC-approved protocols. The Rs1-exon1 knockout (Rs1-KO) was generated by our lab in Long Evans rat using CRISPR/Cas9 by deleting Exon 1, with assistance from Horizon Discovery (Saint Louis, MO, USA) [13]. Long Evans Rs1KO and wild-type (WT) rats (Charles River Laboratories) were used to establish a breeding colony. The breeding colony is maintained at UC Davis Teaching and Research Animal Care Services (TRACS) animal husbandry facility. Using our protocol, genotyping was performed from rat toe clippings at Transnetyx, Inc. (Cordova, TN, USA).

### 2.2. Ocular Tissue Collection and Processing

The rats were sacrificed by carbon dioxide asphyxiation. Their eyes were oriented, enucleated, and fixed with 97% methanol/3% glacial acetic acid for up to 5 days prior to embedding in paraffin [27]. Sagittal sections 5 µm thick were cut through the eye and stained with hematoxylin and eosin (H&E). Retinal images were collected using a Nikon Eclipse e800 microscope with a DS-Ri1 digital camera (Nikon, Tokyo, Japan). For retinal flatmounts, eyes were collected, dissected, fixed with 4%PFA, and kept in 100% MeOH at −20 °C long term. 

### 2.3. Histology and Immunohistochemistry

Tissue sections for morphology examination were deparaffinized in xylene and rehydrated followed by hematoxylin and eosin (H&E) staining. Paraffin sections for immunohistochemistry were rehydrated before blocking, then washed with phosphate buffer 0.1% Tween 20 (1xPBST) and preincubated with serum (5% normal goat, and 0.1% Tween 20 in 1xPBS) at RT for 2 h. Primary antibodies with appropriate dilution using a blocking buffer containing 1xPBS were added and incubated overnight (Table 1). 

The fluorescent secondary antibodies (Alexa Flour 488 goat anti-mouse, Alexa Fluor 568 goat anti-mouse, Alexa Fluor 488 goat anti-chicken, and Alexa Flour 568 goat anti-rabbit; Invitrogen, Carlsbad, CA, USA)) at 1:1000 in PBST were added to retinal sections and incubated for 1.5 h (Table 2). 

Tissue sections were washed in PBST 3 times for 15 min each, cover-slipped with DAPI Fluoromount-G^®^ (SouthernBiotech, Birmingham, AL USA, Cat#: 0100-20) mounting media, and imaged on a confocal laser Scanning Module LSM 510 Microscope System (Carl Zeiss Microscopy, Jena, Germany) and an Olympus Fluoview FV3000 Confocal Laser Scanning Microscope (Olympus, Tokyo, Japan). 

### 2.4. Semi-Quantitative IHC Comparisons

Semi-quantitative IHC comparisons require careful tissue processing, antibody application, and imaging. Sections for comparison across time were processed together for WT and Rs1KO rats in triplicate. IHC comparisons were made between WT and Rs1KO age-matched littermates by breeding Rs1 heterozygous females with WT males, which gave male littermate pups that were either WT Rs1^+/y^ or Rs1KO affected Rs1^−/y^. 

Preliminary studies explored the expression of Aqp4, Kir4.1, vimentin (Vim), glutamine synthetase (GS), glial fibrillary acidic protein (GFAP), isolectin B, and calbindin to establish suitable methods for ocular tissue collection and processing before the final studies were performed. The eyes of the littermates were harvested together and stored in −80 freezer 2–5 days before performing the paraffin embedding process. Then, they were sectioned and placed on slides that were later stored at room temperature. 

At the final stage, all 18 slides (*n* = 3 each, WT and Rs1KO, for P7, P9, and P12) were deparaffinized together, washed, and incubated overnight with antibody aliquots from the same master mix. The slides were scanned at the same time with the same confocal microscope settings. The final images were compared for each set of three eyes to identify the representative images shown in the figures that are presented. For P30, sections of WT and Rs1KO replicates (*n* = 5 for each WT and Rs1KO rat) were performed together to compare IHC following cavity closure. 

### 2.5. Quantification of Aqp4 and Kir4.1 Expression Intensity

The expression levels of Aqp4 and Kir4.1 channels were analyzed quantitatively using ImageJ software (version 2.14, National Institutes of Health, Bethesda, MD, USA). For consistency, three slides each for WT and Rs1KO rats were examined for each time point, and average intensity values were subsequently calculated for statistical comparison. The Polygonal Tool within ImageJ was employed to accurately delineate the retinal boundaries for intensity measurement. The retinal contour was traced manually, extending from the ILM to the retinal pigmented epithelium (RPE). For slides stained with Aqp4 or Kir4.1 antibodies, color channels were individually split to isolate the pertinent signal within the defined retinal boundary region previously defined on the merged image. They were then precisely copied and applied to these isolated channels, ensuring that the analysis remained within the defined retinal regions. The mean gray values, indicative of the expression intensity, were measured.

### 2.6. Statistical Analysis

Data were analyzed using Prism 8 (version 8.02, GraphPad Software, La Jolla, CA, USA) and presented as mean ± SEM. Group comparisons were made with an unpaired *t*-test with Welch’s correction. A *p*-value less than 0.05 was considered significant.

## 3. Results

### 3.1. Small Intraretinal Schisis Cavities Begin to Form by P12 in the Inner Retina

MGCs span the thickness of the retina and envelop retinal neurons in vertebrates. This arrangement facilitates interactions between neurons and MGCs, such as regulating the balance of ions, water, neurotransmitters, and pH levels in the retinal extracellular environment. MGCs can influence angiogenesis control and regulate blood flow in vascularized retinas. Figure 1 is a schematic of MGCs in the retina and the relationship of intermediary filaments with capillary beds. 

**Figure 2:**
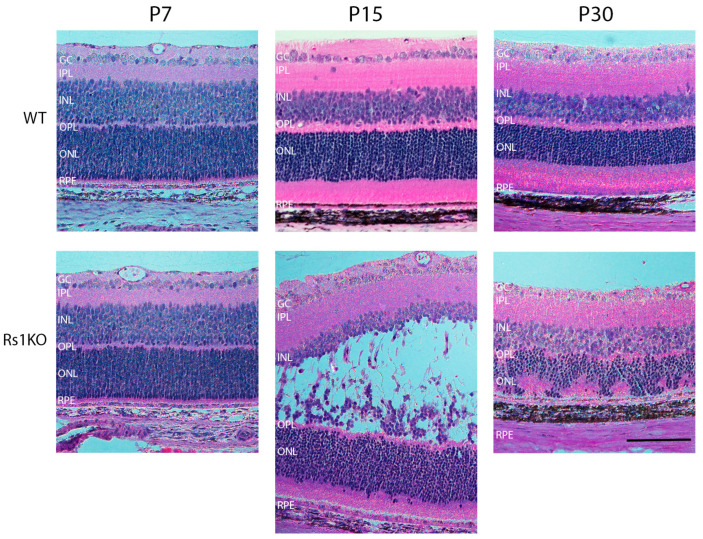
Retinal schisis occurrence in the rat XLRS animal model. The tissue section of the WT and Rs1KO rat retinas stained with H&E at three time points, showing retinal schisis. H&E stain (hematoxylin and eosin) primarily highlights the nuclei of cells in a blue-purple color while staining the cytoplasm and extracellular matrix a pinkish hue. Time points presented are P7, P15, and P30, in wild type (WT) and Rs1 knockout (Rs1KO). GC, ganglion cells; IPL, inner plexiform layer; INL, inner nuclear layer; OPL, outer plexiform layer; ONL, outer nuclear layer; RPE, retinal pigment epithelium (scale bar = 100 µm).

The retinal morphology was analyzed with H&E staining at postnatal timepoints P7, P10, P12, P15, P19, and P30 of Rs1KO and WT rats [13]. At P10, no difference was observed in the morphology of WT and Rs1KO retinas by light microscopy. However, by P12, the Rs1KO rat model exhibited small, discernible intraretinal schisis cavities within the central region of the inner nuclear layer (INL), in the area where MGC soma are located (Figure 2). These cavities enlarged rapidly over the next three days, and by P15, they significantly altered the structural integrity of the INL [13]. 

### 3.2. Developmental Changes in Aqp4 Expression and Distribution 

**Figure 3:**
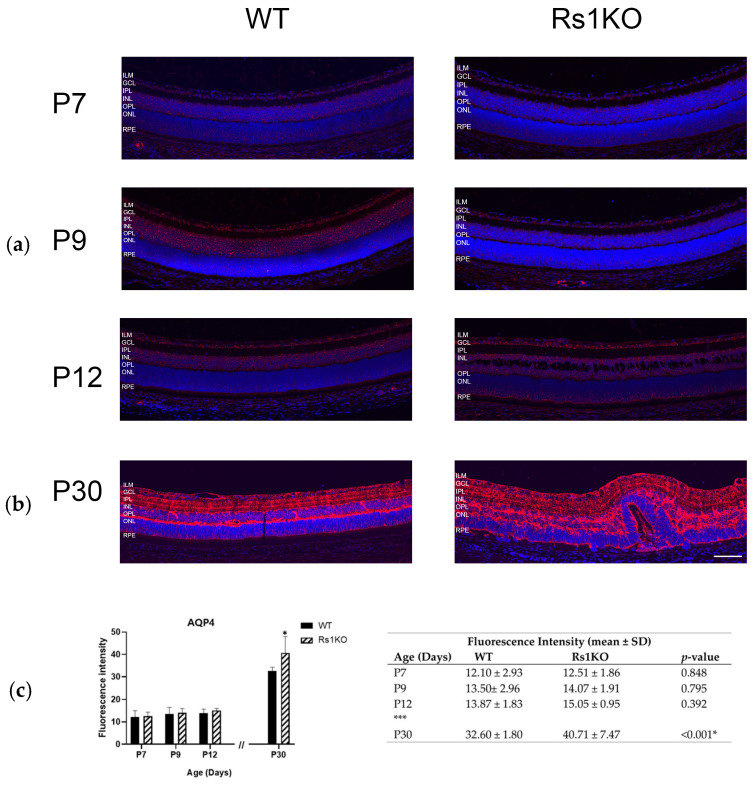
Aquaporin 4 expression and distribution in retinal tissue. Tissue section of WT and Rs1KO rat retinas with Aqp4 fluorescence immunolocalization at various time points: (**a**) preschisis (P7, P9, and P12) and (**b**) postschisis (P30). Confocal images show Aqp4 (red) and the nuclear marker DAPI (blue) (**c**) Expression levels of Aqp4 were analyzed quantitatively using ImageJ software (NIH, Bethesda, MD, USA) in WT and Rs1KO rats: bar graph (left); table (right). The number of replicas was *n* = 3 for each group (WT and Rs1KO) for P7, P9, and P12, and *n* = 5 for each group (WT and Rs1KO) for P30. Rs1KO, knockout; SD, standard deviation; WT, wild type. * Statistically significant. *** indicate time gap between P12 (preschisis) and P30 (postschisis). ILM, inner limiting membrane; GCL, ganglion cell layer; IPL, inner plexiform layer; INL, inner nuclear layer; OPL, outer plexiform layer; ONL, outer nuclear layer; RPE, retinal pigment epithelium (scale bar = 100 µ).

**Figure 4:**
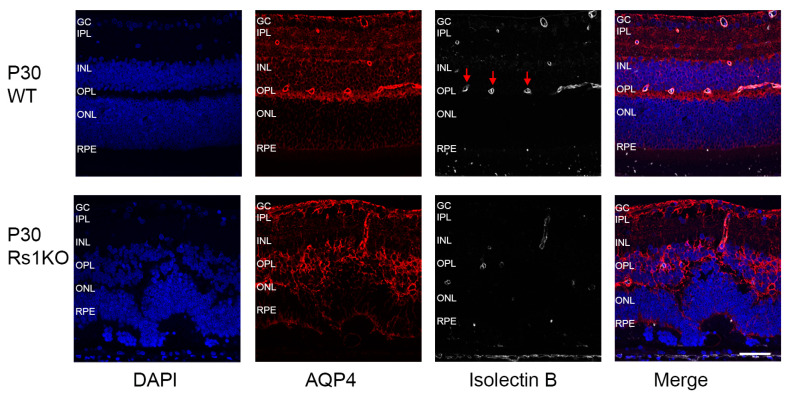
Colocalization of Aqp4 expression with perivascular vessels at P30 in WT and Rs1KO rat retinas. The blood vessels (Isolectin B GS-IB4-silver) and Aqp4 (red) are expressed in MC tightly around blood vessels in WT, but in Rs1KO rat retinas, the expression is much more distributed. Confocal images show Aqp4 (in red), the endothelial cell marker Isolectin B, GS-IB4 (silver), and the nuclear marker DAPI (blue). Aqp4 was concentrated in the perivascular (arrows) and inner limiting membrane domains in the WT animals (scale bar = 50 µ).

In WT retinas, Aqp4 is concentrated at MGC soma and perivascular regions, and at MGC endfeet, Aqp4 is concentrated at the ILM [28]. Aqp4 imaging of WT and Rs1KO retinas showed minimal differences in expression and distribution by or before P12 when the schisis cavities first appeared (Figure 3). However, this changed in the Rs1KO retinas after P15, with the structural tissue disruption caused by the substantial INL cavities, followed by the rapid disappearance of cavities by P19. By P30, when the INL schisis cavities had fully consolidated and disappeared and the tissue was reformed, distortions of the retinal layers remained, and ONL thinning showed a substantial loss of photoreceptors; Aqp4 showed further upregulation of expression and redistribution throughout the Rs1KO retinas. Immunofluorescence colocalization of Aqp4 with vascular endothelial cells (isolectin B GS-IB4) in the WT rats showed that Aqp4 was expressed tightly around blood vessels in MGCs at postnatal timepoints P7, P9, P12, and P30. Aqp4 distribution in P30 Rs1KO rats was more widespread than in WT rats, and perivascular Aqp4 staining expanded to a broader region along the entire length of the MGCs across the Rs1KO retinas (Figure 4). The Aqp4 staining was greatest at P30 versus younger ages (Figure 3). 

Overall, IHC analysis did not implicate changes in Aqp4 expression by P12 as the cavities formed. However, after cavity formation at P15, followed by their collapse, Aqp4 expression was considerably greater in Rs1KO than WT retinas, including along the deep capillary plexus vessels at P30 (Figure 3), consistent with removal of excess extracellular fluid from the inner retina and re-establishing homeostasis. 

### 3.3. Kir 4.1 Expression Is Reduced in Rs1KO 

**Figure 5:**
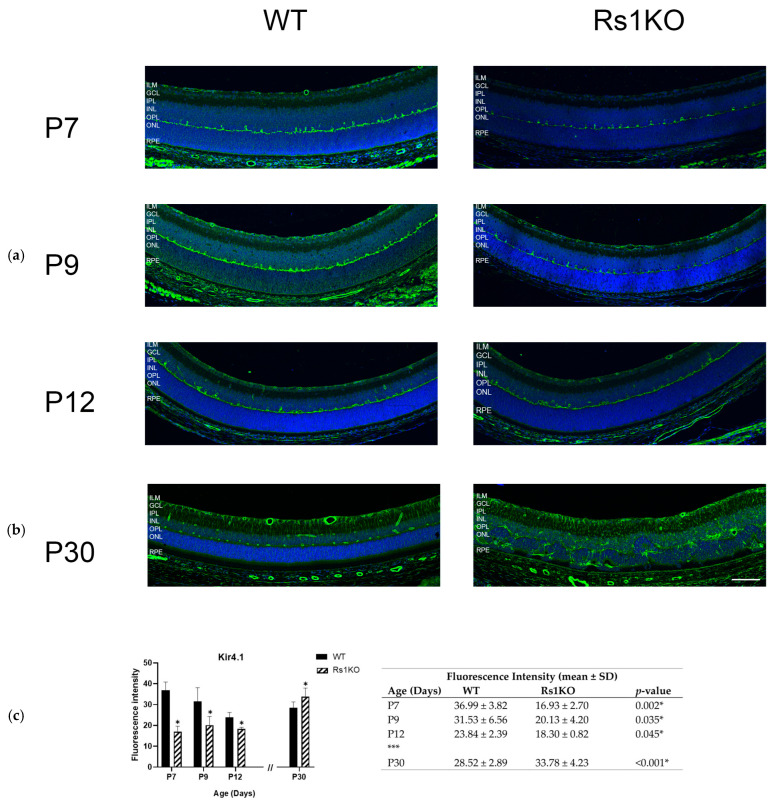
Kir4.1 expression and distribution in rat retinal tissues. Tissue section of WT and Rs1KO rat retinas with Kir4.1 fluorescence immunolocalization at various time points: (**a**) preschisis (P7, P9, and P12) and (**b**) postschisis (P30). Confocal images show Kir4.1 (green) and the nuclear marker DAPI (blue) (**c**) The expression levels of Kir4.1 were analyzed quantitatively using ImageJ software (NIH, Bethesda, MD, USA) in WT and Rs1KO rats: bar graph (left); table (right). The number of replicas was *n* = 3 for each group (WT and Rs1KO) for P7, P9, and P12, and *n* = 5 for each group (WT and Rs1KO) for P30. * Statistically significant. *** indicated time gap between P12 (preschisis) and P30 (postschisis). ILM, inner limiting membrane; GCL, ganglion cell layer; IPL, inner plexiform layer; INL, inner nuclear layer; OPL, outer plexiform layer; ONL, outer nuclear layer; RPE, retinal pigment epithelium (scale bar = 100 µ).

In WT retinas, Kir4.1 expression is seen at the ILM and RPE by P7 and also at the interface of the INL and OPL. This distribution persists at P9 and P12. Expression in the Rs1KO retinas is also found at these locations at P7–P12 but at lower levels then WT retinas. Expression levels do not increase appreciably across the ages of P7–P12 for either WT or Rs1KO retinas, but across all these ages, expression in Rs1KO retinas is considerably less than WT retinas at P30 (Figure 5).

Two points can be noted: at P7 and P9, in both WT and Rs1KO rats, Kir4.1 expression at the OPL has small protrusions into the INL (Supplementary Figure S1, white arrows) that counterstain with calbindin antibody, which labels horizontal cells [29]. The pattern of horizontal cell labeling is progressively less obvious at P12, and by P30, the Kir4.1 localizes along vessels in the DCP and also along MGC processes in the INL and IPL and at the ILM endfeet. This transition from Kir4.1 expression by horizontal cells at early ages and then moving to MGCs at P12–P15 was noted in mice by Bosco lab [30], as also shown here for both WT and Rs1KO rats. 

Second, Kir4.1 labeling is seen at the retinal pigment epithelium (RPE) in WT rats as was described by Kusaka in 1999 [31]. In frogs, Kir4.1 participates in potassium transport from the subretinal space into the choroid [32,33]. This moves water from the distal retina through the RPE, maintaining dehydration of the outer retina [34,35]. The consequence of reduced Kir4.1 expression at both the ILM and RPE would cause a gross disturbance of potassium movement from the retina into the vitreous and the choroid, with a net reduction in water movement out of the retina as the K^+^ concentration gradient is reduced at both locations. 

Newman, 1984, described a process termed “potassium siphoning” into the vitreous through Kir4.1 channels located at the ILM, which moves potassium out of the retina, and water will follow this concentration gradient [19]. These figures show a paucity of Kir4.1 at the ILM/vitreous interface in the Rs1KO rats throughout these ages, consistent with a reduction in K^+^ siphoning into the vitreous and hence a concomitant reduction in water movement out of the retina at the ILM.

### 3.4. Disruption of the Deep Capillary Plexus 

**Figure 6:**
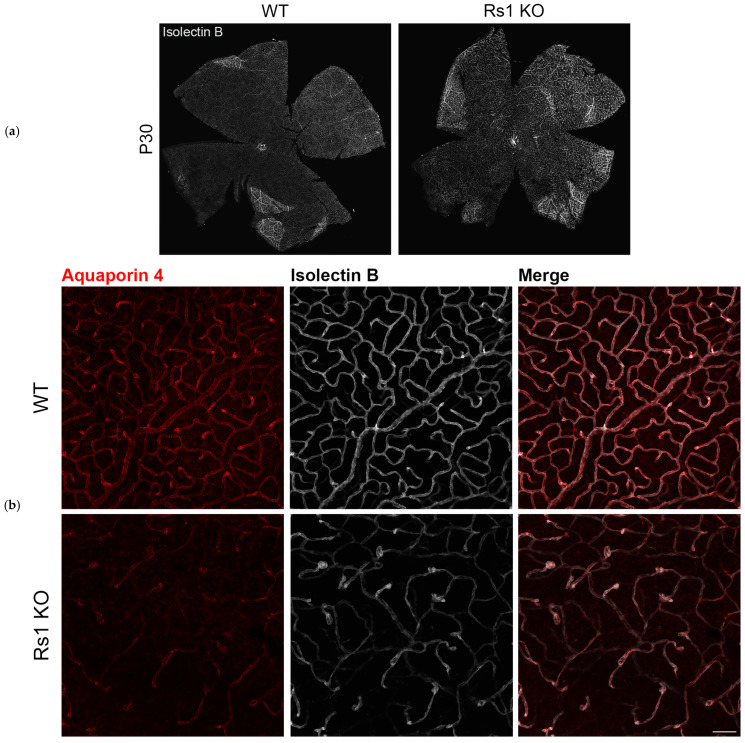
Disruption and thinning of the deep capillary plexus. (**a**) Retinal flatmounts of WT and Rs1KO rats at P30, with blood vessels labeled for isolectin B (GS-IB4 antibody, silver). (**b**) Enlarged regions of retinal flatmounts of WT and Rs1KO rats at P30, with blood vessels labeled for isolectin B (GS-IB4 antibody, silver). Aqp 4 (red) showed DCP disruption associated with MGC perivascular processes (scale bar = 60 µ).

The inner retina of rats has a highly organized three-tiered structure of capillary beds—the superficial, intermediate, and deep capillary plexi (DCP)—very similar to the human retina [36] and serving the metabolic needs of each retinal layer [17]. We imaged the three plexi using retina flatmounts with blood vessels labeled for isolectin B (GS-IB4 antibody) and found disruption of the DCP associated with MGC perivascular processes in Rs1KO rats at P30 (Figure 6) after resolution of the schisis structural disruption of the Rs1KO retinas. At P30, residual vascular pathology is evident, including telangiectatic terminals at the ends of truncated vessels. Aqp4 expression on the DCP vessels in Rs1KO was reduced compared to WT, which showed robust coexpression of isolectin B GS-IB4 and Aqp4. This pathology would be expected to alter MGCs function of water transport in Rs1KO rats through the retina and maintenance of the inner blood–retinal barrier (iBRB) of the inner retina [37].

### 3.5. MGC Expression of Structural Vimentin and Activation Marker GFAP

**Figure 7:**
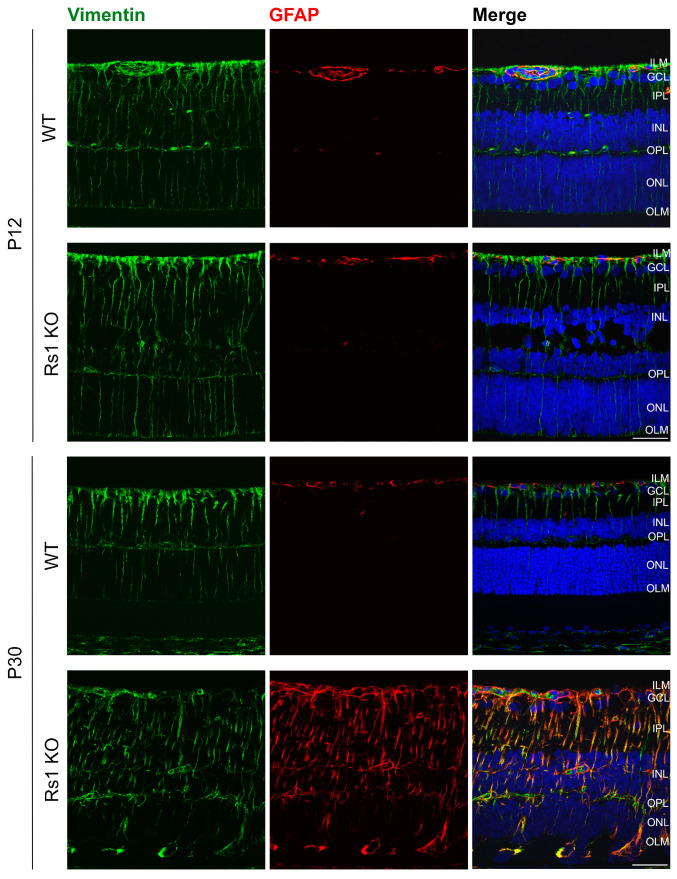
Expression of the MGC marker vimentin and activation marker GFAP. Presented are P12 and P30 time points, both WT and Rs1KO rats. The expression of GFAP and the activation of Muller cells are not present at P12 but are very visible at P30. The number of replicas was n = 3 for each group (WT and Rs1KO) for P12 and P30. Confocal images show GFAP, glial fibrillary activation protein (red), Vim, vimentin (green), and the nuclear marker DAPI (blue). ILM, inner limiting membrane; GCL, ganglion cell layer; IPL, inner plexiform layer; INL, inner nuclear layer; OPL, outer plexiform layer; ONL, outer nuclear layer; OLM, outer limiting membrane (scale bar = 40 µ).

**Figure 8:**
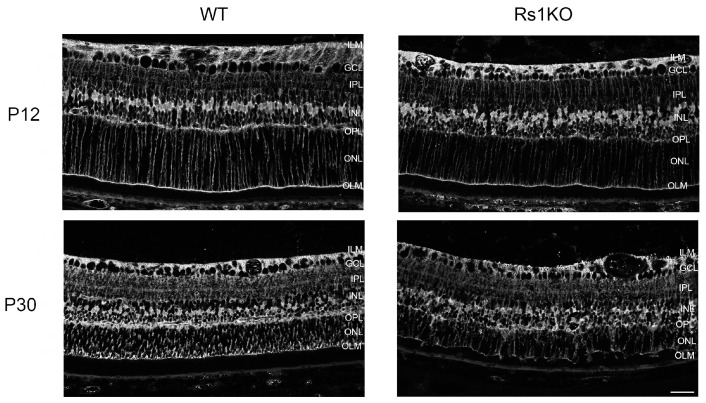
Glutamine synthetase (GS) (silver), exclusively expressed in Muller glia in the retina at P12 and P30, showed the variation in Rs1KO retina MGC soma and nuclei placement, which was spread out and more disorganized in P30 than in P12 images (scale bar = 20 µ).

We examined vimentin (Vim) intermediate filaments and GFAP expression at P12, roughly before schisis formation, and at P30, after cavity resolution (Figure 7). Vim plays a structural role in MGCs and, along with GFAP, contributes to mechanical stabilization and hypertrophy of reactive MGCs during retinal degeneration [38]. At P12, as the cavities were developing, there was no apparent difference in Vim expression in Rs1KO versus WT rats. MGCs spanned the retina normal, with no evident MGC swelling of radial processes as might occur if MGC water homeostasis were disturbed [14]. GFAP expression also showed no differences between WT and Rs1KO retinas at P12 despite the new formation of schisis cystic cavities. However, this had changed by P30, when MGC activation morphology was evident. Similar morphological changes were found in MGC radial processes with glutamine synthetase (GS) antibody at P30, while the MGCs appeared normal at P12 (Figure 8).

## 4. Discussion

This study evaluated whether the appearance of fluid-filled schisis cavities in Rs1KO transgenic rats at P12 correlated with the dysfunctional homeostatic regulation of water flux in the retina by the principal water (Aqp4) and K^+^ (Kir4.1) channels. We found that in the days up to the first appearance of schisis cavities, retinal Kir4.1 expression was reduced at P7, P9, and P12 compared with the WT littermate controls. Kir4.1 is a primary component in ion and water movement from the extracellular space into MGCs and extrusion into the capillary plexus [39]. A reduction in Kir4.1 channels would result in fluid accumulation in the extracellular space and contribute to schisis cavity formation. Kir4.1 channel dysregulation also fits with the retinal appearance of some XLRS individuals, which manifests a Mizuo-Nakamura phenomenon in light, with a “gold leaf” fundus reflex that fades with time and is believed to result from accumulation of extracellular potassium [40]. 

Kir4.1 dysregulation is implicated in other retinal disease processes that result in intraretinal cystic cavities. Diabetic retinopathy (DR) shows the formation of cysts from macular edema with extracellular fluid accumulation in interstitial spaces [41,42]. Kir4.1 downregulation in DR leads to MGC edema and apoptosis [43]. A cascade of events in DR includes the accumulation of advanced glycation end-products (AGEs) as found in other degenerative diseases including atherosclerosis, chronic kidney disease, and Alzheimer’s disease. The accumulation of AGEs causes further decreases in Kir4.1 expression and disrupts MGC activity [44]. Proinflammatory cytokine TNF-α is elevated in DR, which suppresses Kir4.1 expression by reducing its colocalization with synapse-associated protein (SAP97) and causing actin cytoskeleton disorganization [45]. 

Oxidative stress impacts Kir4.1 regulation [46], as do inflammatory mediators, and vascular leakage, leading to macular edema [25]. A presumptive mechanism is that Kir4.1 downregulation causes MGCs to fail in releasing ions into the vasculature, leading to MGCs’ accumulation of K^+^, which leads to osmotic influx of water and MGC swelling [47]. These findings underscore the centrality of proper regulation of Kir4.1 in maintaining retinal health and implicate a number of molecular pathways in Kir4.1 downregulation in the retina. However, a direct connection to RS1 expression has not been identified to date. 

Kir4.1 is also expressed by the RPE [31], where it participates in K^+^ transport from the subretinal space to the choroid, as shown in frogs [32,33]. The RPE is important in facilitating water transport driven by these osmotic ionic gradients [34,48]. Fluid transport across the RPE shows a net movement of water from the outer retina to the choroid in amphibian models [49]. Finding a reduction in Kir4.1 expression at the RPE of Rs1KO mice will perturb the removal of water from the distal retina. 

Disturbing the K^+^ concentration in the subretinal space will diminish light-evoked changes in the photoreceptor’s circulating dark current. This would account for the considerable loss in ERG dark-adapted rod-driven a-wave amplitude observed in this RS1-exon1-rat XLRS model, in which the a-wave loss is disproportionate to loss of photoreceptor cells [13]. We also note that XLRS mouse models with several forms of Rs1 mutations frequently show reduced dark-adapted rod a-wave amplitudes, including in our Rs1 exon-1-del XLRS mouse [19,50,51,52,53,54,55]. Kir4.1 expression is also increased slightly at P30 when it is participating in restoring retinal water homeostasis. 

### 4.1. Aqp4 Expression

Aqp4 is the major water channel in the retina [56]. Unlike Kir4.1, Aqp4 expression in Rs1KO rats was unchanged from WT retinas, with little Aqp4 expressed at ages P7, P9, and P12. In the absence of a change from WT retinas, Aqp4 appears not to be involved directly in schisis cavity formation at P12 in Rs1KO retinas. However, during the subsequent period when excess water was cleared from the retina after large schisis cavities had occurred at P15, Aqp4 expression was upregulated at P30. Hence, Aqp4 is likely involved in reducing schisis cavities and restoring retinal lamination, as seen at P30, albeit with retinal structural distortions as the cavities collapse and also from distortions from irregular loss of photoreceptors in the ONL. Aqp4 expression in the WT rat P30 retinas is focused around areas of MGCs’ contact with capillaries, while in Rs1KO rats at P30, expression is delocalized and spread out across the entire retinal thickness, all the way to photoreceptor’s outer segments and RPE. Hyperosmotic stress can also reduce Aqp4 expression in RPE cells, further hindering water clearance [57].

### 4.2. MGCs’ Involvement 

Kir4.1 is expressed by MGCs, which are central in maintaining retinal homeostasis by regulating ion and water transport [58,59]. Kir4.1 was identified in rat retinas by Ishii et al. (1997) and is associated with MGC endfeet at the ILM vitreous junction [19] and at MGC perivascular processes that wrap around retinal blood vessels [20,21]. Changes in Kir4.1 expression can occur from events that activate MGCs in response to retinal damage. Expression is upregulated during retinal stress injury and indicates a transition from resting to reactive states, a process known as gliosis [60]. GFAP is a marker for MGC activation and is upregulated in various pathological conditions, including diabetic retinopathy, retinopathy of prematurity, and glaucomatous retinal ganglion cell death [61]. 

We looked at GFAP expression in MGCs during the first appearance of schisis cavities, but GFAP expression remained normal at P12 in Rs1KO rats, and the MGC architecture of the extended radial processes was not disturbed, indicating that MGC swelling is unlikely to be a principal component in initial cavity formation. Consequently, the Kir4.1 change appears to be at a regulatory level rather than consequent to MGC swelling. We note that MGCs do not express retinoschisin [62,63], and the connection between the loss of RS1 protein and Kir4.1 remains to be learned.

GFAP was greatly increased in Rs1KO rats by P30, and MGC radial processes were swollen and hypertrophic. A reasonable hypothesis is that MGC activation reflects the failure of homeostatic K^+^ ion and water mechanisms. During normal rat retina development, Kir4.1 channel expression increases in MGCs, which helps regulate MGC volume under conditions of varying osmolarity [64]. However, Kir4.1 expression failed to increase across those times in the Rs1KO rat retinas, and this likely contributes to the MGC swelling and hypertrophy at P30. Also, during mouse postnatal retinal development, Aqp4 and Kir4.1 expression begins in horizontal cells and then switches to Muller glial cells by P15 [30]. The same pattern occurs in rats, as we see horizontal cell expression of Kir4.1 in both WT and Rs1KO rat retinas at P9 by co-labeling with calbindin and Kir4.1 antibodies (Supplementary Figure S1). The schisis cavity formation at P12 and subsequent swelling at P15 occur during this critical period of a change in the source of the Kir4.1 channels. 

### 4.3. Vascular Abnormalities in XLRS Rats

The Rs1KO retinas showed a major disturbance of the deep capillary bed at P30, visualized by labeling retinal flatmounts with isolectin B for blood vessels and MGCs with Aqp4. The DCP plays a crucial role in retinal health [65,66], as DCP disturbance is observed in various retinal diseases. DCP flow density is decreased significantly even in the early stages of human diabetic retinopathy [67]. DCP nonperfusion in diabetic macular ischemia is associated with photoreceptor structural abnormalities and reduced retinal sensitivity, possibly indicating a role in maintaining photoreceptor health [68]. Human XLRS patients also show vascular impairment in the DCP, particularly in the foveal region [5], which correlates with functional outcomes such as limiting visual acuity. However, the DCP changes in the Rs1KO rats were noted particularly at P30 after resolution of the major cystic cavity formation at P15. No change in the DCP was noted in Rs1KO rats at P12 and consequently is not implicated in the first cavity formation. 

### 4.4. Is There a Link Between Rs1 and Kir4.1? 

These findings implicate the reduced expression of Kir4.1 during early development in the chain of pathology leading to schisis cavities. The finding also implicates Aqp4 in the re-establishment of intraretinal fluid homeostasis later at P30 as cavities disappear. While both Kir4.1 and Aqp4 are expressed by MGCs, we have not established a positive link between RS1 expression and events involving Kir4.1 and Aqp4. 

Current understanding does not indicate that MGCs express RS1, so this possibly easy link is missing. RS1 protein is expressed by retinal neurons at several levels, including by photoreceptors and bipolar cells [8,69]. It is secreted and bound to the outer plasma membrane of photoreceptor inner segments [7,8,70]. Following secretion, RS1 remains at the outer membrane surface and facilitates interactions and adhesion among photoreceptor cells, bipolar cells, and MGCs, and these interactions are compromised when RS1 is mutated and nonfunctional [11,71]. 

Retinoschisin also has a role in stabilizing membrane-associated channels or ligands on neural membranes. Interacting partners include the ATP1A3 and ATP1B2 subunits of retinal Na/K-ATPase on the plasma membrane of photoreceptor inner segments [72,73]. Retinoschisin interacts with voltage-gated potassium (Kv) channel subunits Kv2.1 and Kv8.2 [74,75]. Retinoschisin binds to L-type voltage-gated calcium channels (LTCCs) Cav1.3 and Cav1.4, which are involved in calcium influx and photoreceptor neurotransmitter release [75]. Although RS1 is important for anchoring or coordinating membrane proteins including ion channels, interactions with Kir4.1 have not been noted. 

RS1 deficiency leads to the mislocalization of the macromolecular complex and reduces the expression of interacting protein partners, although it does not affect the K^+^ currents mediated by Kv channels [72]. Retinoschisin is involved in regulating ERK signaling and apoptosis in retinal cells [75]. It is also involved in MAP kinase signaling pathways and downstream targets [76]. As ERK and MAP kinase signaling are core elements in many cellular processes, it is possible that RS1 may influence the regulation of the expressions of other proteins like Aqp4 and Kir4.1, directly or indirectly. 

### 4.5. Water Flux and Accumulation in the Retina 

There is net water flow across the retina from the vitreous to the choriocapillaris, with several intraretinal sources and sinks [34]. In the proximal retina, water enters primarily through the vitreous at the retinal surface and from the retinal vasculature with the blood–retinal barrier (BRB). As discussed above, extracellular water is taken up in the proximal retina by MGCs with Aqp4 and Kir4.1 channels and extruded into the retinal vasculature. In the distal retina, water is extruded through the RPE by active K^+^ transport through Kir4.1 [31,77]. This flow is countered with a reverse flow back into the distal retina through RPE paracellular junctions [78,79,80]. 

Excess fluid would accumulate at the structurally weakest part of the retina, which appears to be the middle of the INL, at least by evidence of fluid accumulation in other retinal pathologies. Human diabetic macular edema occurs preferentially in the middle layer of the retina and across the macula, which mirrors the location of schisis cavities in XLRS disease [81]. Retinal inflammatory processes also result in macular fluid accumulation in the INL [41]. And the rare genetic disease Familial Exudative Vitreoretinopathy (FEVR) also shows fluid accumulation at this retinal level [82]. These findings point toward some structural weakness in the middle of the INL, which is predisposed to fluid accumulation. And as the XLRS retina lacks RS1 as an “adhesion protein” [10,63], this would cause additional structural weakness contributing to accumulation. Of interest, careful OCT observations of human preterm infants without RS1 disease show that intraretinal cystic fluid accumulates in the INL beginning at 33 weeks of gestation in about one-third of cases, further indicating some anatomical weakness in this INL region [83]. 

### 4.6. Limitations of This Study

There are potential limitations of the rat model and applying it across multiple species including humans. The rat is a nocturnal animal with retinas that are more rod-dominated than those of humans, and the rat does not have a macula. We have studied two XLRS rat models, including this one [13,84], and both models show greatly diminished ERG a-waves that are not commonly observed in humans. The rat model also differs from the XLRS mouse model in this regard. However, this study provides a starting basis for considering water and abnormal water movement contributing to schisis formation in XLRS rats.

## 5. Conclusions

It is important to recognize that this XLRS rat model has two distinct periods of XLRS pathology: (1) an onset phase during early-developmental times leading to initial schisis cavity formation at P12 and (2) the cavity resolution phase in mid- to late development, when presumably homeostatic mechanisms react to the excess fluid accumulation and cause cavity closure.

- Kir4.1 expression was lower in Rs1KO rats during cavity onset at P7, P9, and P12, while Aqp4 expression remained at WT rat levels and is not implicated in schisis cavity formation at P12.
- No overt MGC structural changes were noted by P12, nor did MGCs express GFAP by P12. Hence, the Kir4.1 changes were at a regulatory level and not the result of MGC activation.
- Both Aqp4 and Kir4.1 expressions were strongly upregulated in Rs1KO rats throughout the retina by P30 during fluid removal and resolution of the cavities.
- At P30, MGCs also showed increased GFAP reactivity, and radial processes were hypertrophic.
- The intraretinal vascular networks were disturbed by P15, after major schisis formation and collapse (Figure 2 and [13]). The DCP capillary pattern was sparse at the INL/OPL border. Human XLRS patients show similar changes in the DCP [5]. Hence, the alteration of the capillary vascular network appears reactive but not causative for schisis cavity formation at P12.
- As MGCs are not believed to express RS1 protein, while both Kir4.1 and Aqp4 are associated with MGCs, the causal link of pathology remains uncertain. This leads to the question of whether Rs1 expression by retinal neurons helps stabilize the retinal extra cellular matrix or leads to the indirect action of Rs1 with MGC membranes, which keeps the whole system structurally stable and functionally integrated.

### 5.1. Our Current Understanding 

We started on the question of abnormal water accumulation in Rs1KO retinas by looking for a single “smoking gun”, using semi-quantitative IHC to assess Aqp4 and Kir4.1. Indeed, Kir4.1 is considerably reduced in the Rs1KO retinas by and before P12 when the schisis cavities first appear. However, additional events that contribute to the process also happen. INL and OPL vascularization changes as the DCP forms by P12. OLM breakdown begins by P12–P15 as photoreceptor cells migrate from the ONL, through the OLM, and into the RIS/ROS region [13]. The OLM is a semi-barrier composed of MGC processes joined to the inner segments at the sub-apical region with a tight junction complex; OLM failure will increase water entry into the outer retina from the choriocapillaris [85]. By P30, the ILM shows thinning, and water extrusion that accompanies “potassium syphoning” through Kir4.1 is likely altered. 

Our current thinking is that the schisis pathology results from a complex interplay of these multiple factors during the initial stages of P12 cavity formation, which is followed by a rapid massive increase in cavity size at P15 and then cavity collapse and resolution by P30. The first stage at P12 includes reduced retinal Kir4.1 expression, which alters osmotic gradients, while impaired Kir4.1 function at the RPE leads to outer retina fluid imbalance, which collects in the INL. The ILM and OLM are both disrupted at about this time, and this leads to further failure of fluid homeostasis by P15. This is followed by the upregulation of Aqp4 and Kir4.1, which plays its role in re-establishing (possibly incomplete) homeostasis by removing fluid and the collapse of cavities. These multiple events collectively lead to the pathological accumulation of water in the inner retina, resulting in macular edema and some degree of associated visual impairment.

### 5.2. Significance of Our Research

Our results are significant for the elucidation of the complex underlying pathology of the XLRS and for identifying new therapeutic targets. Gene therapy and optogenetics are two promising approaches for treating X-linked retinoschisis (XLRS). Gene therapy aims to correct the underlying genetic defect [86,87,88,89], while optogenetics seeks to restore vision by introducing light-sensitive proteins into retinal cells [90]. Both strategies have shown potential in preclinical studies, but they face distinct challenges and opportunities in clinical outcomes. Both approaches continue to evolve, with ongoing research aimed at overcoming these hurdles and improving clinical outcomes.

## Figures and Tables

**Table 1:** Primary antibodies with appropriate dilution used in fluorescent immunohistology.

Antibody	Species	Vendor	Cat#	Dilution
Aquaporin 4 (Aqp4)	Rabbit	Alomone Labs	AQP-004	1:500
Inwardly rectifying K^+^ channel (Kir4.1)	Rabbit	Alomone Labs	APC-035	1:1000
Glutamine synthetase (GS)	Mouse	Millipore Sigma	MA5-27750	1:500
Glial fibrillary acidic protein (GFAP)	Mouse	Cell Signaling Technology	3670T	1:500
Vimentin (Vim)	Chicken	Custom Ab, FitzGerald Lab		1:1000
Isolectin B, GS-IB4, AF-647-conjugated		Invitrogen	I32450	1:500
Calbindin	Mouse	Millipore Sigma	C9848	1:500

**Table 2:** Fluorescent secondary antibodies with appropriate dilution used in fluorescent immunohistology.

Second Antibody	Vendor	Cat#	Dilution
Goat anti-mouse, AF405-conjugated	Invitrogen	A-48255	1:1000
Goat anti-mouse, AF568-conjugated	Invitrogen	A-11004	1:1000
Goat anti-chicken, AF488-conjugated	Invitrogen	A-32931	1:1000
Goat anti-rabbit, AF568-conjugated	Invitrogen	A-11011	1:1000

## Data Availability

All the generated data are presented in this paper.

## Supplementary Materials

The following supporting information can be downloaded at https://www.mdpi.com/article/10.3390/genes15121583/s1, Figure S1: Colocalization of calbindin and Kir4.1 in WT and Rs1KO rat retinas at age P9. Retinal horizontal cells (calbindin-AF488); Kir4.1channel (AF568) 20X. NFL, nerve fiber layer; GCL, ganglion cell layer; IPL, inner plexiform layer; INL, inner nuclear layer; OPL, outer plexiform layer; ONL, outer nuclear layer; OLM, outer limiting membrane; RPE, retinal pigment epithelium (scale bar = 50 µm).

## References

[1] Fahim A.T., Ali N., Blachley T., Michaelides M. (2017). Peripheral fundus findings in X-linked retinoschisis. Br. J. Ophthalmol..

[2] Rao P., Dedania V.S., Drenser K.A. (2018). Congenital X-Linked Retinoschisis: An Updated Clinical Review. Asia Pac. J. Ophthalmol..

[3] Chen D., Xu T., Tu M., Xu J., Zhou C., Cheng L., Yang R., Yang T., Zheng W., He X. (2017). Recapitulating X-Linked Juvenile Retinoschisis in Mouse Model by Knock-In Patient-Specific Novel Mutation. Front. Mol. Neurosci..

[4] Kwon H.J., Kim Y.N., Min C.H., Kim Y.J., Lee J., Lee J.Y., Yoon Y.H. (2022). MACULAR MICROVASCULATURE IN X-LINKED RETINOSCHISIS: Optical Coherence Tomography and Optical Coherence Tomography Angiography Study. Retina.

[5] Romano F., Arrigo A., Ch’ng S.W., Battaglia Parodi M., Manitto M.P., Martina E., Bandello F., Stanga P.E. (2019). Capillary Network Alterations in X-Linked Retinoschisis Imaged on Optical Coherence Tomography Angiography. Retina.

[6] Audo I., Mohand-Saïd S., Sahel J.A., Holder G.E., Moore A.T., Puech B., De Laey J.J., Holder G.E. (2014). X-Linked Retinoschisis. Inherited Chorioretinal Dystrophies.

[7] Molday R.S. (2007). Focus on molecules: Retinoschisin (RS1). Exp. Eye Res..

[8] Molday L.L., Wu W.W., Molday R.S. (2007). Retinoschisin (RS1), the protein encoded by the X-linked retinoschisis gene, is anchored to the surface of retinal photoreceptor and bipolar cells through its interactions with a Na/K ATPase-SARM1 complex. J. Biol. Chem..

[9] Heymann J.B., Vijayasarathy C., Huang R.K., Dearborn A.D., Sieving P.A., Steven A.C. (2019). Cryo-EM of retinoschisin branched networks suggests an intercellular adhesive scaffold in the retina. J. Cell Biol..

[10] Tolun G., Vijayasarathy C., Huang R., Zeng Y., Li Y., Steven A.C., Sieving P.A., Heymann J.B. (2016). Paired octamer rings of retinoschisin suggest a junctional model for cell-cell adhesion in the retina. Proc. Natl. Acad. Sci. USA.

[11] Vijayasarathy C., Takada Y., Zeng Y., Bush R.A., Sieving P.A. (2008). Organization and molecular interactions of retinoschisin in photoreceptors. Adv. Exp. Med. Biol..

[12] Ou J., Vijayasarathy C., Ziccardi L., Chen S., Zeng Y., Marangoni D., Pope J.G., Bush R.A., Wu Z., Li W. (2015). Synaptic pathology and therapeutic repair in adult retinoschisis mouse by AAV-RS1 transfer. J. Clin. Investig..

[13] Ye E.A., Zeng Y., Thomas S., Sun N., Smit-McBride Z., Sieving P.A. (2022). XLRS Rat with Rs1(-/Y) Exon-1-Del Shows Failure of Early Postnatal Outer Retina Development. Genes.

[14] Navneet S., Wilson K., Rohrer B. (2024). Muller Glial Cells in the Macula: Their Activation and Cell-Cell Interactions in Age-Related Macular Degeneration. Investig. Ophthalmol. Vis. Sci..

[15] Bringmann A., Pannicke T., Grosche J., Francke M., Wiedemann P., Skatchkov S.N., Osborne N.N., Reichenbach A. (2006). Muller cells in the healthy and diseased retina. Prog. Retin. Eye Res..

[16] Reichenbach A., Bringmann A. (2020). Glia of the human retina. Glia.

[17] Srinivasan V.J., Moshiri A. (2020). Imaging oxygenation of retinal capillaries with depth resolution. Proc. Natl. Acad. Sci. USA.

[18] Orkand R.K., Nicholls J.G., Kuffler S.W. (1966). Effect of nerve impulses on the membrane potential of glial cells in the central nervous system of amphibia. J. Neurophysiol..

[19] Newman E.A., Frambach D.A., Odette L.L. (1984). Control of extracellular potassium levels by retinal glial cell K^+^ siphoning. Science.

[20] Nagelhus E.A., Horio Y., Inanobe A., Fujita A., Haug F.M., Nielsen S., Kurachi Y., Ottersen O.P. (1999). Immunogold evidence suggests that coupling of K^+^ siphoning and water transport in rat retinal Muller cells is mediated by a coenrichment of Kir4.1 and AQP4 in specific membrane domains. Glia.

[21] Kofuji P., Biedermann B., Siddharthan V., Raap M., Iandiev I., Milenkovic I., Thomzig A., Veh R.W., Bringmann A., Reichenbach A. (2002). Kir potassium channel subunit expression in retinal glial cells: Implications for spatial potassium buffering. Glia.

[22] Djukic B., Casper K.B., Philpot B.D., Chin L.S., McCarthy K.D. (2007). Conditional knock-out of Kir4.1 leads to glial membrane depolarization, inhibition of potassium and glutamate uptake, and enhanced short-term synaptic potentiation. J. Neurosci..

[23] Kucheryavykh Y.V., Kucheryavykh L.Y., Nichols C.G., Maldonado H.M., Baksi K., Reichenbach A., Skatchkov S.N., Eaton M.J. (2007). Downregulation of Kir4.1 inward rectifying potassium channel subunits by RNAi impairs potassium transfer and glutamate uptake by cultured cortical astrocytes. Glia.

[24] Riepe R.E., Norenberg M.D. (1978). Glutamine synthetase in the developing rat retina: An immunohistochemical study. Exp. Eye Res..

[25] Reichenbach A., Wurm A., Pannicke T., Iandiev I., Wiedemann P., Bringmann A. (2007). Muller cells as players in retinal degeneration and edema. Graefes Arch. Clin. Exp. Ophthalmol..

[26] Smit-McBride Z., Thomas S.R., Sun N., Sieving P.A. Aquaporin-4 and Kir4.1 channels in post-natal pathology of retinoschisis in Rs1 exon-1 del rat. Abstract in Association for Research in Vision and Ophthalmology (ARVO) 2023 Meeting. 2023: New Orleans, Louisiana.

[27] Sun N., Shibata B., Hess J.F., FitzGerald P.G. (2015). An alternative means of retaining ocular structure and improving immunoreactivity for light microscopy studies. Mol. Vis..

[28] Katoozi S., Rao S.B., Skauli N., Froehner S.C., Ottersen O.P., Adams M.E., Amiry-Moghaddam M. (2020). Functional specialization of retinal Muller cell endfeet depends on an interplay between two syntrophin isoforms. Mol. Brain.

[29] Mitchell C.K., Rowe-Rendleman C.L., Ashraf S., Redburn D.A. (1995). Calbindin immunoreactivity of horizontal cells in the developing rabbit retina. Exp. Eye Res..

[30] Bosco A., Cusato K., Nicchia G.P., Frigeri A., Spray D.C. (2005). A developmental switch in the expression of aquaporin-4 and Kir4.1 from horizontal to Muller cells in mouse retina. Investig. Ophthalmol. Vis. Sci..

[31] Kusaka S., Horio Y., Fujita A., Matsushita K., Inanobe A., Gotow T., Uchiyama Y., Tano Y., Kurachi Y. (1999). Expression and polarized distribution of an inwardly rectifying K^+^ channel, Kir4.1, in rat retinal pigment epithelium. J. Physiol..

[32] Immel J., Steinberg R.H. (1986). Spatial buffering of K^+^ by the retinal pigment epithelium in frog. J. Neurosci..

[33] Kofuji P., Ceelen P., Zahs K.R., Surbeck L.W., Lester H.A., Newman E.A. (2000). Genetic inactivation of an inwardly rectifying potassium channel (Kir4.1 subunit) in mice: Phenotypic impact in retina. J. Neurosci..

[34] Marshall A.T., Crewther S.G. (2022). Osmotic gradients and transretinal water flow-a quantitative elemental microanalytical study of frozen hydrated chick eyes. Front. Cell. Neurosci..

[35] Bringmann A., Uckermann O., Pannicke T., Iandiev I., Reichenbach A., Wiedemann P. (2005). Neuronal versus glial cell swelling in the ischaemic retina. Acta Ophthalmol. Scand..

[36] Burns S.A., Elsner A.E., Gast T.J. (2021). Imaging the Retinal Vasculature. Annu. Rev. Vis. Sci..

[37] Pigaiani N., Bertaso A., De Palo E.F., Bortolotti F., Tagliaro F. (2020). Vitreous humor endogenous compounds analysis for post-mortem forensic investigation. Forensic Sci. Int..

[38] Ostrowska-Podhorodecka Z., McCulloch C.A. (2021). Vimentin regulates the assembly and function of matrix adhesions. Wound Repair. Regen..

[39] Fujita A., Inanobe A., Hibino H., Nielsen S., Ottersen O.P., Kurachi Y. (2015). Clustering of Kir4.1 at specialized compartments of the lateral membrane in ependymal cells of rat brain. Cell Tissue Res..

[40] Wakabayashi K., Sakai-Wakabayashi Y., Ishigami C. (2022). Mizuo-Nakamura phenomenon in X-linked retinoschisis. Am. J. Ophthalmol. Case Rep..

[41] Haydinger C.D., Ferreira L.B., Williams K.A., Smith J.R. (2023). Mechanisms of macular edema. Front. Med..

[42] Lai D., Wu Y., Shao C., Qiu Q. (2023). The Role of Muller Cells in Diabetic Macular Edema. Investig. Ophthalmol. Vis. Sci..

[43] Li X., Lv J., Li J., Ren X. (2021). Kir4.1 may represent a novel therapeutic target for diabetic retinopathy (Review). Exp. Ther. Med..

[44] Thompson K., Chen J., Luo Q., Xiao Y., Cummins T.R., Bhatwadekar A.D. (2018). Advanced glycation end (AGE) product modification of laminin downregulates Kir4.1 in retinal Muller cells. PLoS ONE.

[45] Hassan I., Luo Q., Majumdar S., Dominguez J.M., Busik J.V., Bhatwadekar A.D. (2017). Tumor Necrosis Factor Alpha (TNF-α) Disrupts Kir4.1 Channel Expression Resulting in Muller Cell Dysfunction in the Retina. Investig. Ophthalmol. Vis. Sci..

[46] Luo Q., Xiao Y., Alex A., Cummins T.R., Bhatwadekar A.D. (2019). The Diurnal Rhythm of Insulin Receptor Substrate-1 (IRS-1) and Kir4.1 in Diabetes: Implications for a Clock Gene Bmal1. Investig. Ophthalmol. Vis. Sci..

[47] Pannicke T., Iandiev I., Wurm A., Uckermann O., vom Hagen F., Reichenbach A., Wiedemann P., Hammes H.P., Bringmann A. (2006). Diabetes alters osmotic swelling characteristics and membrane conductance of glial cells in rat retina. Diabetes.

[48] Miller S.S., Hughes B.A., Machen T.E. (1982). Fluid transport across retinal pigment epithelium is inhibited by cyclic AMP. Proc. Natl. Acad. Sci. USA.

[49] Bissig D., Berkowitz B.A. (2012). Light-dependent changes in outer retinal water diffusion in rats in vivo. Mol. Vis..

[50] Marangoni D., Wu Z., Wiley H.E., Zeiss C.J., Vijayasarathy C., Zeng Y., Hiriyanna S., Bush R.A., Wei L.L., Colosi P. (2014). Preclinical safety evaluation of a recombinant AAV8 vector for X-linked retinoschisis after intravitreal administration in rabbits. Hum. Gene Ther. Clin. Dev..

[51] Park T.K., Wu Z., Kjellstrom S., Zeng Y., Bush R.A., Sieving P.A., Colosi P. (2009). Intravitreal delivery of AAV8 retinoschisin results in cell type-specific gene expression and retinal rescue in the Rs1-KO mouse. Gene Ther..

[52] Kjellstrom S., Bush R.A., Zeng Y., Takada Y., Sieving P.A. (2007). Retinoschisin gene therapy and natural history in the Rs1h-KO mouse: Long-term rescue from retinal degeneration. Investig. Ophthalmol. Vis. Sci..

[53] Takada Y., Fariss R.N., Muller M., Bush R.A., Rushing E.J., Sieving P.A. (2006). Retinoschisin expression and localization in rodent and human pineal and consequences of mouse RS1 gene knockout. Mol. Vis..

[54] Zeng Y., Takada Y., Kjellstrom S., Hiriyanna K., Tanikawa A., Wawrousek E., Smaoui N., Caruso R., Bush R.A., Sieving P.A. (2004). RS-1 Gene Delivery to an Adult Rs1h Knockout Mouse Model Restores ERG b-Wave with Reversal of the Electronegative Waveform of X-Linked Retinoschisis. Investig. Ophthalmol. Vis. Sci..

[55] Liu Y., Kinoshita J., Ivanova E., Sun D., Li H., Liao T., Cao J., Bell B.A., Wang J.M., Tang Y. (2019). Mouse models of X-linked juvenile retinoschisis have an early onset phenotype, the severity of which varies with genotype. Hum. Mol. Genet..

[56] Gleiser C., Wagner A., Fallier-Becker P., Wolburg H., Hirt B., Mack A.F. (2016). Aquaporin-4 in Astroglial Cells in the CNS and Supporting Cells of Sensory Organs-A Comparative Perspective. Int. J. Mol. Sci..

[57] Willermain F., Janssens S., Arsenijevic T., Piens I., Bolaky N., Caspers L., Perret J., Delporte C. (2014). Osmotic stress decreases aquaporin-4 expression in the human retinal pigment epithelial cell line, ARPE-19. Int. J. Mol. Med..

[58] Yandiev Y., Bringmann A., Wiedemann P. (2012). Role of retinal glial cells in pathogenesis of macular oedema. Acta Ophthalmol..

[59] Goodyear M.J., Crewther S.G., Junghans B.M. (2009). A role for aquaporin-4 in fluid regulation in the inner retina. Vis. Neurosci..

[60] Sanchez M.C., Chiabrando G.A. (2022). Multitarget Activities of Muller Glial Cells and Low-Density Lipoprotein Receptor-Related Protein 1 in Proliferative Retinopathies. ASN Neuro.

[61] Tabasi A., Ghafari S., Mehdizadeh M., Shekari M.A., Golalipour M.J. (2017). Gestational diabetes influences retinal Muller cells in rat’s offspring. Iran. J. Basic. Med. Sci..

[62] Byrne L.C., Ozturk B.E., Lee T., Fortuny C., Visel M., Dalkara D., Schaffer D.V., Flannery J.G. (2014). Retinoschisin gene therapy in photoreceptors, Muller glia or all retinal cells in the Rs1h-/- mouse. Gene Ther..

[63] Reid S.N., Yamashita C., Farber D.B. (2003). Retinoschisin, a photoreceptor-secreted protein, and its interaction with bipolar and muller cells. J. Neurosci..

[64] Wurm A., Pannicke T., Iandiev I., Wiedemann P., Reichenbach A., Bringmann A. (2006). The developmental expression of K^+^ channels in retinal glial cells is associated with a decrease of osmotic cell swelling. Glia.

[65] Chiaravalli G., Guidoboni G., Sacco R., Radell J., Harris A. (2022). A multi-scale/multi-physics model for the theoretical study of the vascular configuration of retinal capillary plexuses based on OCTA data. Math. Med. Biol..

[66] Thanos A., Young J., Fortune B., Tang S.J. (2023). The retinal deep capillary plexus as a venous outflow system; insights from Sturge Weber Syndrome. Retin. Cases Brief. Rep..

[67] Kaizu Y., Nakao S., Arima M., Wada I., Yamaguchi M., Sekiryu H., Hayami T., Ishikawa K., Ikeda Y., Sonoda K.H. (2019). Capillary dropout is dominant in deep capillary plexus in early diabetic retinopathy in optical coherence tomography angiography. Acta Ophthalmol..

[68] Scarinci F., Varano M., Parravano M. (2019). Retinal Sensitivity Loss Correlates with Deep Capillary Plexus Impairment in Diabetic Macular Ischemia. J. Ophthalmol..

[69] Takada Y., Fariss R.N., Tanikawa A., Zeng Y., Carper D., Bush R., Sieving P.A. (2004). A retinal neuronal developmental wave of retinoschisin expression begins in ganglion cells during layer formation. Investig. Ophthalmol. Vis. Sci..

[70] Vijayasarathy C., Takada Y., Zeng Y., Bush R.A., Sieving P.A. (2007). Retinoschisin is a peripheral membrane protein with affinity for anionic phospholipids and affected by divalent cations. Investig. Ophthalmol. Vis. Sci..

[71] Molday L.L., Hicks D., Sauer C.G., Weber B.H., Molday R.S. (2001). Expression of X-linked retinoschisis protein RS1 in photoreceptor and bipolar cells. Investig. Ophthalmol. Vis. Sci..

[72] Schmid V., Wurzel A., Wetzel C.H., Plossl K., Bruckmann A., Luckner P., Weber B.H.F., Friedrich U. (2022). Retinoschisin and novel Na/K-ATPase interaction partners Kv2.1 and Kv8.2 define a growing protein complex at the inner segments of mammalian photoreceptors. Cell Mol. Life Sci..

[73] Plossl K., Straub K., Schmid V., Strunz F., Wild J., Merkl R., Weber B.H.F., Friedrich U. (2019). Identification of the retinoschisin-binding site on the retinal Na/K-ATPase. PLoS ONE.

[74] Shi L., Ko M.L., Ko G.Y. (2017). Retinoschisin Facilitates the Function of L-Type Voltage-Gated Calcium Channels. Front. Cell. Neurosci..

[75] Plossl K., Royer M., Bernklau S., Tavraz N.N., Friedrich T., Wild J., Weber B.H.F., Friedrich U. (2017). Retinoschisin is linked to retinal Na/K-ATPase signaling and localization. Mol. Biol. Cell.

[76] Plossl K., Weber B.H., Friedrich U. (2017). The X-linked juvenile retinoschisis protein retinoschisin is a novel regulator of mitogen-activated protein kinase signaling and apoptosis in the retina. J. Cell Mol. Med..

[77] Steinberg R.H. (1985). Interactions between the retinal pigment epithelium and the neural retina. Doc. Ophthalmol..

[78] Ghassemifar R., Lai C.M., Rakoczy P.E. (2006). Regulation of tight junction proteins in cultured retinal pigment epithelial cells and in VEGF overexpressing transgenic mouse retinas. Adv. Exp. Med. Biol..

[79] Dvoriashyna M., Foss A.J.E., Gaffney E.A., Repetto R. (2020). Fluid and solute transport across the retinal pigment epithelium: A theoretical model. J. R. Soc. Interface.

[80] Bora K., Kushwah N., Maurya M., Pavlovich M.C., Wang Z., Chen J. (2023). Assessment of Inner Blood-Retinal Barrier: Animal Models and Methods. Cells.

[81] Romero-Aroca P., Baget-Bernaldiz M., Pareja-Rios A., Lopez-Galvez M., Navarro-Gil R., Verges R. (2016). Diabetic Macular Edema Pathophysiology: Vasogenic versus Inflammatory. J. Diabetes Res..

[82] Tanenbaum R., Acon D., Rodriguez A., Negron C., Berrocal A. (2021). Macular Retinal Pigment Epithelial Clumping Leading to a Diagnosis of FEVR. Ophthalmic Surg. Lasers Imaging Retin..

[83] Maldonado R.S., O’Connell R., Ascher S.B., Sarin N., Freedman S.F., Wallace D.K., Chiu S.J., Farsiu S., Cotten M., Toth C.A. (2012). Spectral-domain optical coherence tomographic assessment of severity of cystoid macular edema in retinopathy of prematurity. Arch. Ophthalmol..

[84] Zeng Y., Qian H., Campos M.M., Li Y., Vijayasarathy C., Sieving P.A. (2021). Rs1h(-/y) exon 3-del rat model of X-linked retinoschisis with early onset and rapid phenotype is rescued by RS1 supplementation. Gene Ther..

[85] Omri S., Omri B., Savoldelli M., Jonet L., Thillaye-Goldenberg B., Thuret G., Gain P., Jeanny J.C., Crisanti P., Behar-Cohen F. (2010). The outer limiting membrane (OLM) revisited: Clinical implications. Clin. Ophthalmol..

[86] Mishra A., Sieving P.A. (2021). X-linked Retinoschisis and Gene Therapy. Int. Ophthalmol. Clin..

[87] Thompson D.A., Iannaccone A., Ali R.R., Arshavsky V.Y., Audo I., Bainbridge J.W.B., Besirli C.G., Birch D.G., Branham K.E., Cideciyan A.V. (2020). Advancing Clinical Trials for Inherited Retinal Diseases: Recommendations from the Second Monaciano Symposium. Transl. Vis. Sci. Technol..

[88] Vijayasarathy C., Sardar Pasha S.P.B., Sieving P.A. (2021). Of men and mice: Human X-linked retinoschisis and fidelity in mouse modeling. Prog. Retin. Eye Res..

[89] Vijayasarathy C., Zeng Y., Brooks M.J., Fariss R.N., Sieving P.A. (2021). Genetic Rescue of X-Linked Retinoschisis Mouse (Rs1(-/y)) Retina Induces Quiescence of the Retinal Microglial Inflammatory State Following AAV8-RS1 Gene Transfer and Identifies Gene Networks Underlying Retinal Recovery. Hum. Gene Ther..

[90] Simunovic M.P., Shen W., Lin J.Y., Protti D.A., Lisowski L., Gillies M.C. (2019). Optogenetic approaches to vision restoration. Exp. Eye Res..

