# Supplementary figures and images for: Kir4.1 and Aqp4 Contribution to Schisis Cystic Water Accumulation and Clearance in the Rs1 Exon-1 Del XLRS Rat Model
Source: Genes (Basel). 2024 Dec 9;15(12):1583. doi: 10.3390/genes15121583 (PMC11675908; doi:10.3390/genes15121583)

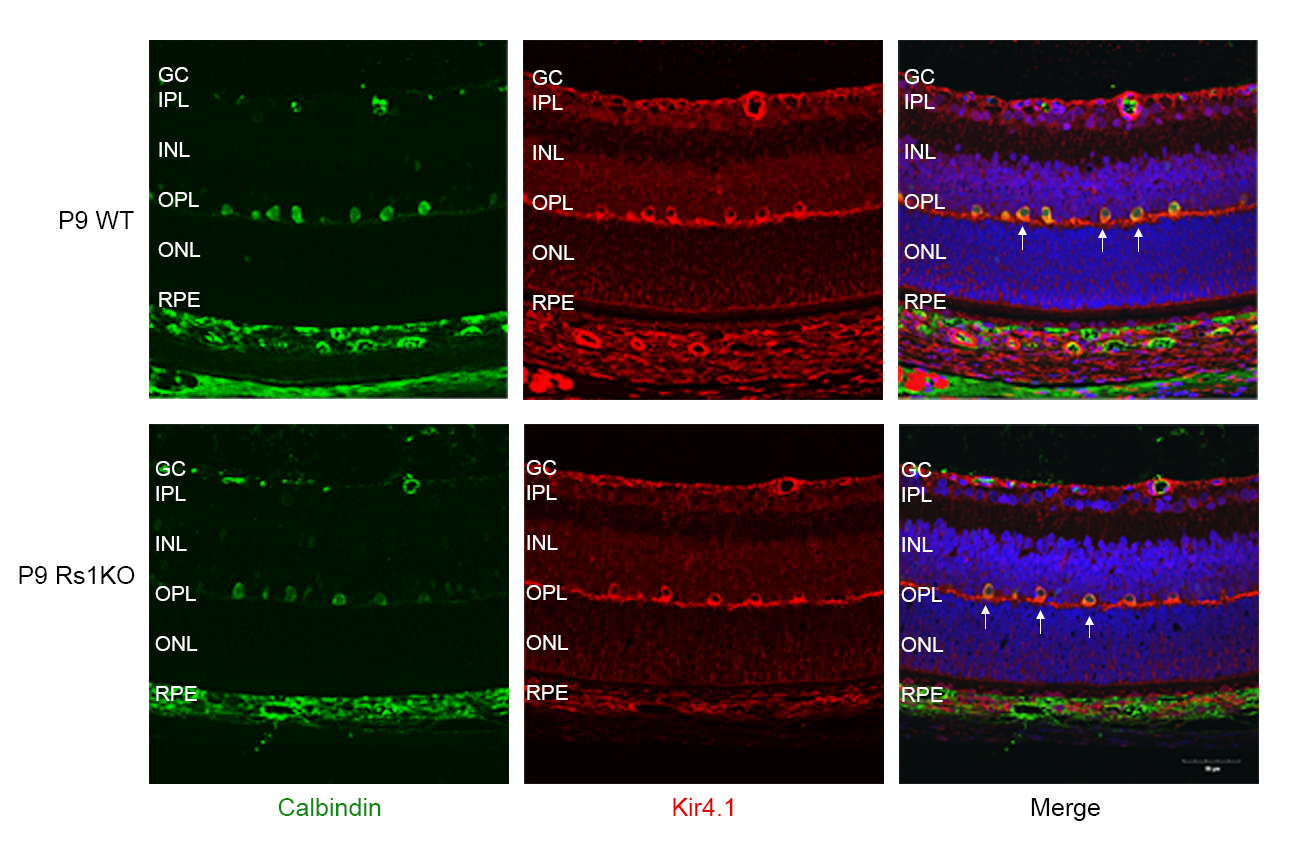

Supplement: Supplementary file 1 [file genes-15-01583-s001.zip › genes-3301348-supplementary.tif]
